# Supplementary material for: Uncertainty-aware personalized estimation of Parkinson’s disease severity from longitudinal speech
Source: PLoS One. 2026 Aug 5;21(8):e0343191. doi: 10.1371/journal.pone.0343191 (PMC13440870; doi:10.1371/journal.pone.0343191)
Supplement: Supplementary file 1 [file pone.0343191.s001.pdf]

Response to the Reviewers

Reviewer 1

Comment 1: Quantum of work is less.

Response:

I thank the reviewer for this important observation. I agree that several individual components used in the proposed framework, such as recurrent temporal modeling and latent variable inference, have appeared separately in prior literature. However, the primary contribution of the present work lies in the integration of these components into a unified uncertainty-aware longitudinal telemonitoring framework for Parkinson's disease severity estimation. In the revised manuscript, the evaluation criteria are made more rigorous, and several additional experiments are performed. Please refer to the revised manuscript.

Comment 2: Its a popular dataset which consists of pre-calculated parameters and this work is another statistical interpretation.

Response:

I thank the reviewer for this important observation. I acknowledge that the Oxford Parkinson's telemonitoring dataset is a widely used benchmark dataset and that the acoustic dysphonia measures are precomputed rather than learned directly from raw speech signals. The primary objective of the present study is therefore not acoustic feature engineering or representation discovery, but rather the development and analysis of an uncertainty-aware longitudinal modeling framework for estimating Parkinson's disease severity. The dataset was selected because it remains **one of the few publicly available longitudinal Parkinson's speech datasets containing repeated motor UPDRS assessments**, enabling reproducible comparison with prior telemonitoring studies. I agree that reliance on precomputed features limits the scope of conclusions regarding low-level acoustic biomarker discovery, and I have clarified this limitation explicitly in the revised manuscript.

Comment 3: In a bigger work this can be one way to substantiate the results and considering only this as a big work is not correct in my opinion. Thus the work is limited to a particular dataset and difficult to generalize.

Response:

I thank the reviewer for this important observation and agree that evaluation on a single dataset limits the generalizability of the conclusions. The primary intentions of this present work are not

to claim universal clinical applicability, but rather to investigate the feasibility of uncertainty-aware longitudinal modeling for Parkinson's disease telemonitoring under controlled settings. I acknowledge that the Oxford Parkinson's telemonitoring dataset represents a relatively small and specialized cohort with limited temporal duration and specific recording conditions. Accordingly, I have revised the manuscript to more carefully frame the proposed framework as a methodological proof-of-concept with experimental validations rather than a deployment-ready clinical system.

Comment 4: Its Intellectually good piece of work but more contributions shall be done by considering a dataset with customized parameters of the authors choice and a full complete work shall be tried out. The noise shall be added synthetically to UPDRS and the robustness of the proposed algorithm shall be tested.

Response:

I thank the reviewer for the constructive suggestion. I agree that validation on larger and more heterogeneous datasets, including datasets with customized or newly derived speech representations, would further strengthen the contribution and broader applicability of the proposed framework. However, the current work was primarily intended as a methodological study of uncertainty-aware longitudinal modeling using one of the few publicly available Parkinson's telemonitoring datasets containing repeated motor UPDRS assessments.

To address the reviewer's recommendation regarding robustness evaluation, I conducted additional experiments in which synthetic Gaussian noise was added to the motor UPDRS labels during training (Please refer to section 5.6 and Table 6). The results demonstrated gradual performance degradation as the noise level increased, while the framework retained relatively stable predictive behavior under moderate perturbation levels. These findings suggest that the proposed probabilistic longitudinal framework exhibits reasonable robustness to imperfect or noisy clinical supervision.

The revised manuscript now includes both quantitative results and a discussion of this robustness analysis, along with a more explicit acknowledgment of the limitations associated with the current dataset and feature representation (Please refer to the discussion section).

Reviewer 2

Comment 1: Extremely high predictive performance and risk of overfitting. The reported performance is exceptionally high, particularly the near-perfect explained variance and very low prediction error. While the authors acknowledge dataset characteristics, these values raise concerns regarding potential overfitting or implicit leakage through temporal or subject-specific structure. The dataset consists of repeated longitudinal measurements with low short-term variability, which may inflate performance. The manuscript would benefit from a more critical and quantitative analysis of this issue. Specifically, please provide comparison with naive baselines such as last observation carried forward or subject-specific mean prediction, performance under more challenging splits such as reduced history or temporal extrapolation, and ablation of patient embeddings to assess how much performance depends on subject identity rather than disease signal.

Response:

I thank the reviewer for this important and insightful observation. I agree that the initially reported predictive performance is exceptionally high and that the longitudinal structure of the dataset, including repeated measurements with relatively low short-term variability, may introduce strong temporal continuity and subject-specific effects that inflate performance. To address this concern more rigorously, I substantially expanded the experimental evaluation in the revised manuscript.

First, I added comparisons with several naive temporal baselines, including global mean prediction, subject-specific mean prediction, and last-observation-carried-forward (LOCF) (Please refer to Table.3 in the revised manuscript). The LOCF baseline achieved particularly strong performance, confirming the presence of substantial short-term temporal autocorrelation within the dataset trajectories. This analysis is now explicitly discussed in the manuscript to better contextualize the reported regression performance.

Second, I conducted additional temporal extrapolation experiments in which the model was trained on earlier trajectory segments and evaluated on later visits (Please refer to section 5.1, last paragraph). Under this substantially more challenging setting, predictive performance decreased compared to the original evaluation but remained reasonably strong, suggesting that the framework captures longer-range disease progression patterns beyond immediate temporal persistence.

Third, I performed limited-history experiments using temporal windows consisting of only one or two prior recordings to assess dependence on longitudinal context (Please refer to section 5.7). While performance improved with larger windows, the model retained stable predictive capability under sparse-history conditions, supporting potential applicability in realistic telemonitoring scenarios with limited prior data.

Finally, I added a component-wise ablation analysis, including the removal of patient-specific embeddings (Please refer to section 5.8). Results showed that patient embeddings contribute meaningfully to predictive performance, confirming that part of the model behavior depends on

subject-specific longitudinal structure. This limitation is now discussed more explicitly in the revised manuscript.

Overall, the manuscript has been revised to more carefully position the proposed framework as an uncertainty-aware longitudinal telemonitoring model evaluated under multiple robustness analyses, rather than as a universally generalizable clinical prediction system.

.

Comment 2: Generalizability and external validity. The study relies on a single dataset with a relatively small cohort and a short observation period. All subjects are unmedicated and recently diagnosed, which significantly limits the generalizability of the findings. The conclusions regarding clinical applicability and telemonitoring potential are therefore somewhat overstated. At minimum, the manuscript should more explicitly acknowledge this limitation and temper claims accordingly. Ideally, validation on an independent cohort or at least a simulated domain shift analysis would strengthen the work.

Response:

I thank the reviewer for this important observation and agree that the use of a single relatively small longitudinal dataset limits the broader generalizability of the findings. The Oxford Parkinson's telemonitoring dataset consists primarily of recently diagnosed and unmedicated patients recorded over a relatively short observation period, which does not fully represent the heterogeneity encountered in real-world clinical populations. Accordingly, I have revised the manuscript to moderate claims regarding clinical applicability and deployment readiness.

To address this concern more rigorously, I added additional robustness analyses in the revised manuscript. Specifically, I conducted temporal extrapolation experiments evaluating performance on later unseen trajectory segments, limited-history experiments using only one or two prior recordings, and **a simulated domain shift analysis using synthetic perturbations of the clinical targets** (Please refer to Table 6 in the revised manuscript). These experiments were intended to assess robustness under more challenging longitudinal conditions beyond the original evaluation setting.

While the proposed framework demonstrated reasonably stable behavior under these additional analyses, I agree that validation on larger independent multi-center cohorts with more heterogeneous disease stages, medication states, and recording conditions will be essential before broader clinical conclusions can be established. This limitation and the need for external validation are now discussed more explicitly in both the Discussion and Conclusion sections.

Comment 3: Personalization strategy and potential information leakage

The use of learnable patient-specific embeddings is an interesting design choice, but it raises conceptual and practical concerns. Since embeddings are learned per subject, it is unclear how the model behaves for truly new patients in real-world deployment. Even though a patient-wise split is used, the mechanism by which embeddings are initialized or adapted for unseen patients is not sufficiently described. This is a critical issue, as personalization may inadvertently encode subject identity rather than disease progression. Please clarify how the model generalizes to new patients without prior embedding information and consider including an analysis without embeddings or with adaptation strategies.

Response:

I thank the reviewer for this important observation. I agree that the use of patient-specific embeddings introduces important considerations regarding generalization to unseen subjects and the potential encoding of subject identity rather than disease-related dynamics. In the original manuscript, the role and limitations of the personalization mechanism were not sufficiently clarified, and this has now been revised.

In the current framework, embeddings are learned only for subjects observed during training and are not adapted online for unseen patients. Consequently, the personalized embeddings primarily function as subject-dependent latent context during longitudinal modeling rather than as a deployable patient initialization strategy for cold-start scenarios. To better assess the dependence of the framework on subject identity information, I conducted an additional ablation experiment in which patient embeddings were completely removed from the model (Please refer to section 5.8).

The ablation results showed a measurable reduction in predictive performance without embeddings, indicating that personalization contributes meaningfully to the learned longitudinal representation. However, the model remained functional even without patient-specific embeddings, suggesting that the temporal speech features themselves still contain predictive disease-related information beyond subject identity alone.

I agree that adaptation strategies for truly unseen patients represent an important future direction. The revised manuscript now discusses possible extensions, including meta-learning, adaptive embedding initialization, and online personalization strategies for real-world deployment settings (Please refer to the discussion section).

Comment 4: Uncertainty quantification and clinical interpretation

The incorporation of uncertainty is a strength of the manuscript, but its clinical usefulness remains insufficiently demonstrated. The results suggest that uncertainty reflects variability but not necessarily prediction error. This limits interpretability in a clinical decision-making context. The manuscript would benefit from more rigorous evaluation of uncertainty calibration, for example, using calibration curves, expected calibration error, or coverage probability of prediction intervals. Without such analysis, it is difficult to assess whether the uncertainty estimates are reliable.

Response:

I thank the reviewer for this important and constructive observation. I agree that visualizing uncertainty alone is insufficient to establish reliability in a clinical context and that more rigorous calibration analysis is necessary to interpret the uncertainty estimates meaningfully.

To address this concern, I added additional uncertainty calibration experiments in the revised manuscript, including prediction interval coverage probability (PICP) analysis and reliability calibration curves after variance scaling calibration (Please refer to section 5.5). The calibrated uncertainty estimates achieved coverage probabilities reasonably close to expected Gaussian confidence levels, indicating partially calibrated global predictive intervals. Reliability calibration analysis further demonstrated generally consistent calibration behavior across multiple confidence levels, although mild under-coverage remained at higher confidence intervals.

At the same time, correlation analysis between predictive uncertainty and sample-wise prediction error remained weak, suggesting that the current latent uncertainty formulation is more effective at providing globally calibrated confidence intervals than identifying locally difficult predictions. Accordingly, I revised the manuscript to moderate claims regarding clinical interpretability and clarified that the reported uncertainty should primarily be interpreted as model confidence rather than clinically validated predictive reliability (Please refer to discussion section).

I agree that more advanced uncertainty modeling and calibration strategies, including heteroscedastic likelihood modeling, conformal prediction, and Bayesian calibration techniques, represent important future directions for improving clinical applicability of uncertainty-aware telemonitoring systems.

Comment 5: Ordinal classification and clinical validity

The ordinal severity labels are derived using quantile-based thresholds rather than clinically established staging systems. While this approach is methodologically convenient, it reduces clinical interpretability and limits comparability with existing literature. The manuscript should discuss this limitation more explicitly and, if possible, provide an additional analysis using clinically meaningful thresholds or better justify the chosen discretization approach.

Response:

I thank the reviewer for this important observation. I agree that the ordinal severity labels used in this study were derived from quantile-based discretization rather than clinically established staging criteria, which limits direct clinical interpretability and comparability with prior clinical literature. The primary motivation for this approach was the relatively limited sample size and imbalanced distribution of motor UPDRS scores within the available dataset, which made construction of stable clinically stratified groups challenging.

I have revised the manuscript to discuss this limitation more explicitly and to clarify that the ordinal severity analysis should be interpreted primarily as a methodological auxiliary supervision strategy rather than a clinically validated staging system. I agree that future work should investigate clinically grounded severity categorization schemes and validation on larger cohorts with sufficient representation across disease stages. Please refer to the discussion section in the revised manuscript.

Comment 6: Comparison with prior work. Although a comparison with prior work is provided, it is not entirely fair due to differences in evaluation protocols, data splits, and preprocessing pipelines. Some prior works report substantially different performance under different conditions. While this is acknowledged, the manuscript still draws strong conclusions regarding superiority. The comparison should be framed more cautiously, and ideally, baseline models should be reimplemented and evaluated under the same experimental conditions.

Response:

I thank the reviewer for this important observation. I agree that direct comparison with previously published results should be interpreted cautiously due to differences in evaluation protocols, preprocessing procedures, temporal splitting strategies, and experimental settings across studies. In the revised manuscript, I have moderated claims regarding superiority and clarified that the reported comparisons are intended primarily for general contextual reference rather than strict head-to-head benchmarking (Please refer to section 5.9).

A complete reimplementation and standardized evaluation of prior methods under identical experimental conditions would certainly provide a more rigorous comparative analysis. However, such an extensive benchmarking study is beyond the current scope of this work, which is primarily focused on investigating uncertainty-aware longitudinal modeling and robustness analysis within a unified framework. This limitation and the need for standardized comparative evaluation are now discussed more explicitly in the manuscript (**Please refer to the discussion section**).

Comment 7: Model complexity versus dataset size. The proposed model combines temporal modeling, latent variable inference, ordinal regression, and personalization. Given the relatively small dataset size, there is a risk that model complexity exceeds what the data can robustly support. The hyperparameter sensitivity analysis suggests stability, but does not fully address overparameterization. An ablation study demonstrating the contribution of each component would significantly strengthen the manuscript.

Response:

I thank the reviewer for this important observation. I agree that the proposed framework combines multiple modeling components and that the relatively small dataset size raises valid concerns regarding potential overparameterization. While the original manuscript included hyperparameter sensitivity analysis, I acknowledge that this alone does not sufficiently establish the contribution of individual components.

To address this concern, I added a comprehensive ablation study evaluating the effect of removing ordinal supervision, probabilistic latent inference, patient-specific embeddings, and temporal modeling individually (Please refer to section 5.8). The results showed that each component contributes positively to predictive performance, with temporal modeling and patient-specific embeddings providing the largest performance gains. In contrast, removal of ordinal supervision and probabilistic latent inference produced smaller but consistent degradations, suggesting that these modules provide complementary regularization effects rather than unnecessary complexity.

Comment 8: Clinical relevance and interpretation of results. While the methodological contribution is clear, the clinical implications are somewhat overstated. The manuscript suggests that the model could support clinical decision-making, yet no analysis is provided regarding clinically meaningful thresholds, progression detection, or treatment-related changes. The discussion should be more cautious and better aligned with what is actually demonstrated, namely, predictive modeling on a specific dataset rather than validated clinical utility.

Response:

I thank the reviewer for this important observation and agree that the original discussion may have overstated the clinical implications of the proposed framework. The current study evaluates predictive modeling performance on a specific longitudinal speech dataset and does not directly assess clinically actionable outcomes such as treatment response, progression detection thresholds, or real-world clinical decision support.

In the revised manuscript, I have moderated claims regarding clinical applicability and clarified that the proposed framework should primarily be interpreted as a methodological investigation of uncertainty-aware longitudinal modeling for speech-based Parkinson's disease assessment. While

the results suggest potential utility for longitudinal telemonitoring, further validation on larger clinical cohorts and evaluation against clinically meaningful endpoints will be necessary before establishing practical clinical utility or deployment readiness. Please refer to the discussion section in the revised manuscript.

Comment 9: Dataset limitations and temporal scope. The dataset spans a relatively short period and shows weak progression trends at the population level. This raises questions about whether the model is truly capturing disease progression or primarily learning short-term temporal consistency and subject-specific baselines. The manuscript would benefit from a more explicit discussion of this limitation and its implications for long-term disease monitoring.

Response:

I thank the reviewer for this important observation and agree that the relatively short temporal duration and limited progression variability of the dataset represent important limitations of the current study. The longitudinal trajectories in the Oxford Parkinson's telemonitoring dataset exhibit relatively weak population-level progression over the available observation period, which increases the likelihood that the model partially relies on short-term temporal continuity and subject-specific baseline characteristics.

To address this concern, I added additional analyses, including temporal extrapolation experiments, naive temporal baseline comparisons, reduced-history evaluation, and embedding ablation studies (Please refer to the changes in the revised manuscript). These experiments demonstrated that temporal continuity contributes substantially to predictive performance, although the model retained reasonably stable behavior under more challenging evaluation settings.

The manuscript has been revised to more explicitly acknowledge that the current results should be interpreted primarily as evidence of longitudinal modeling capability within a constrained telemonitoring setting rather than definitive long-term disease progression modeling. I agree that validation on datasets with longer follow-up duration and stronger progression dynamics will be essential for evaluating applicability to long-term Parkinson's disease monitoring.

Comment 10: The manuscript is generally well written, but certain sections could be streamlined for clarity. Some methodological descriptions are dense and could benefit from simplification. A clearer schematic overview of the model architecture would also improve readability, particularly for readers less familiar with probabilistic modeling approaches.

Response:

I thank the reviewer for the constructive feedback. In the revised manuscript, I simplified several methodological descriptions and reduced unnecessary technical density to improve readability and overall clarity. Additional explanatory text was also added in sections describing the probabilistic temporal modeling framework and uncertainty estimation process.

Furthermore, I revised and improved the schematic architecture figure to provide a clearer overview of the complete modeling pipeline, including temporal encoding, latent probabilistic inference, personalization, ordinal supervision, and uncertainty-aware prediction (Please refer to Fig.3 in the revised manuscript). These revisions were intended to make the framework more accessible to readers who may be less familiar with probabilistic longitudinal modeling approaches.

Reviewer 3

Comment 1: Limitations were noted with the current dataset, including its short duration and including only unmedicated patients. Are there other independent datasets that could be used to validate the model, such as ones with medicated patients?

Response:

I thank the reviewer for this important suggestion. At present, publicly available longitudinal Parkinson's speech datasets containing repeated motor UPDRS assessments, medication variability, and sufficiently dense follow-up recordings remain extremely limited. To the best of my knowledge, there are currently no openly accessible datasets with comparable longitudinal telemonitoring structure that include substantial populations of medicated patients suitable for direct external validation of the proposed framework.

I agree that evaluation on independent cohorts with broader disease heterogeneity, medication effects, and longer follow-up duration would substantially strengthen the clinical relevance and generalizability of the study. This limitation and the need for future validation on larger multi-center datasets are now discussed more explicitly in the revised manuscript (Please refer to the discussion section).

Comment 2: This model can predict UPDRS with longitudinal windows of 10 recordings. How well would the model perform with only one or two recordings? This would be important for real world use with new patients.

Response:

I thank the reviewer for this valuable suggestion. To evaluate performance under limited longitudinal context, I conducted additional experiments using temporal windows consisting of only one or two prior recordings. With a single-recording window, the framework achieved an

MAE of 1.710, RMSE of 2.292, and R^2 = 0.920. Using two recordings further improved performance to an MAE of 1.552, RMSE of 2.087, and R^2 = 0.934. (Please refer to section 5.7)

Although performance improved with longer temporal windows, the model remained reasonably stable even under extremely limited history conditions. These findings suggest that the proposed framework may retain practical utility in real-world telemonitoring scenarios involving newly enrolled patients or sparse longitudinal follow-up data.

Comment 3: Could this model be adapted to predict future UPDRS scores?'

Response:

I thank the reviewer for this insightful suggestion. In principle, the proposed framework could be extended to predict future motor UPDRS scores by reformulating the current next-step longitudinal prediction setting into a forecasting-oriented temporal prediction task. The probabilistic temporal structure and latent disease representation used in the present study are naturally compatible with future trajectory modeling.

Although explicit long-horizon forecasting was beyond the scope of the current work, the additional temporal extrapolation experiments included in the revised manuscript provide preliminary evidence that the framework retains predictive capability on later unseen trajectory segments. Future work will investigate dedicated forecasting formulations for predicting longer-term disease progression and clinically meaningful progression trajectories.

Comment 4: The model is described as uncertainty-aware and incorporates a variance estimate based on Gaussian distribution. While including a variance measure is useful and promising, it is not clear that the variance has been shown to correspond to clinically meaningful uncertainty. How should this variance be interpreted in the clinical setting? For example, does a wider uncertainty range indicate that the UPDRS estimate is less reliable?

Response:

I thank the reviewer for this important observation. I agree that the original manuscript did not sufficiently clarify the interpretation and limitations of the reported uncertainty estimates in a clinical context. In the revised manuscript, I added additional uncertainty calibration analyses, including prediction interval coverage probability and reliability calibration evaluation after variance scaling calibration (Please refer to section 5.5 in the revised manuscript).

The results showed that the calibrated uncertainty intervals achieved reasonably consistent global coverage behavior, suggesting that wider uncertainty intervals generally correspond to lower overall model confidence. However, the correlation between uncertainty magnitude and sample-wise prediction error remained weak, indicating that the current uncertainty formulation should

not yet be interpreted as a fully reliable indicator of local prediction difficulty or direct clinical reliability.

Accordingly, I revised the manuscript to clarify that the reported variance primarily reflects model confidence and latent representation uncertainty rather than clinically validated decision uncertainty. In a practical setting, wider uncertainty intervals may indicate that the model is less certain about the predicted UPDRS estimate, but further calibration and prospective clinical validation would be necessary before such uncertainty measures could support clinical decision-making reliably.

Comment 5: Speech abnormalities are seen in other conditions. Could this impact the specificity of such a model.

Response:

I thank the reviewer for this important observation. I agree that speech abnormalities are not specific to Parkinson’s disease and may also occur in other neurological, respiratory, or age-related conditions. Consequently, the proposed framework should not be interpreted as a diagnostic model for Parkinson’s disease, but rather as a longitudinal severity estimation approach evaluated within a clinically characterized Parkinson’s cohort.

The current study assumes that subjects already have an established PD diagnosis and focuses on tracking disease-related severity patterns over time rather than differentiating PD from other disorders. I agree that evaluation on more heterogeneous cohorts, including subjects with other speech-affecting conditions, would be important for assessing specificity and broader clinical applicability.

Comment 6: Some acronyms were introduced without being expanded first. Please ensure all acronyms are defined.

Response:

I thank the reviewer for pointing this out. In the revised manuscript, I carefully reviewed the text to ensure that all acronyms and abbreviations are expanded at their first occurrence and used consistently throughout the manuscript to improve readability and clarity.

Sincerely,

K. A. Shahriar

Corresponding Author.
