# Supplementary material for: Uncertainty-aware personalized estimation of Parkinson’s disease severity from longitudinal speech
Source: PLoS One. 2026 Aug 5;21(8):e0343191. doi: 10.1371/journal.pone.0343191 (PMC13440870; doi:10.1371/journal.pone.0343191)
Supplement: Supplementary file 2 [file pone.0343191.s002.pdf]

Response to the Reviewers

Reviewer 2

Comment 1: First, although the additional analyses help contextualize the very high predictive performance, the reported R^2 remains extremely high and should continue to be interpreted cautiously. The manuscript should avoid implying that this level of performance would necessarily generalize to broader clinical settings.

Response:

I thank the reviewer for this thoughtful observation. I have revised the manuscript to further soften the claim regarding the reported R^2 values and to avoid implying broad clinical generalizability. I also clarify that these results may not directly generalize to broader clinical settings with more heterogeneous patient populations, longer disease trajectories, and sparser sampling. Corresponding revisions have been made in the Results, Discussion, and Conclusion sections, acknowledging that further validation on larger and more diverse longitudinal cohorts is required before broader clinical applicability can be established. Please refer to the revised manuscript.

Comment 2: Second, the issue of patient-specific embeddings is improved but not fully resolved. The author now clarifies that embeddings are learned for training subjects and are not adapted online for unseen patients. This is an important limitation for real-world deployment and should be emphasized more clearly, particularly in relation to cold-start use in new patients.

Response:

I thank the reviewer for this constructive criticism. I agree that the absence of online adaptation for patient-specific embeddings represents an important limitation for real-world deployment. To address this concern, I have revised the Discussion to explicitly state that patient-specific embeddings are learned only for training subjects and remain fixed for previously unseen patients, resulting in a cold-start personalization scenario for newly enrolled individuals. I further clarify that this limitation may reduce performance in practical longitudinal deployment and identify online adaptation, meta-learning, and adaptive embedding initialization as promising directions for future work to progressively refine patient representations over time. Please refer to the revised manuscript.

Comment 3: Third, the uncertainty analysis is improved, but the finding that uncertainty is weakly associated with sample-wise prediction error limits its clinical interpretability. The

manuscript should clearly state that the current uncertainty estimates are not yet validated as reliable indicators of individual-level clinical prediction reliability.

Response:

I thank the reviewer for this insightful comment. I agree that the weak association between predictive uncertainty and sample-wise prediction error limits the current clinical interpretability of the uncertainty estimates. Accordingly, I have revised the manuscript to explicitly acknowledge this limitation. In both the Results and Discussion sections, the revised manuscript now states that although the proposed probabilistic framework provides reasonably calibrated prediction intervals after post-hoc variance scaling, the current uncertainty estimates should not be interpreted as validated indicators of individual-level clinical prediction reliability. Instead, they are presented as a probabilistic characterization of predictive variability, and I emphasize that further methodological development and validation on larger and more diverse clinical cohorts are required before uncertainty estimates can be used to support individual-level clinical decision-making. Please refer to the revised manuscript.

Comment 4: Fourth, the ordinal severity classification remains based on quantile-derived categories rather than clinically validated staging thresholds. This is acceptable as an auxiliary modeling strategy, but the abstract and discussion should avoid presenting these categories as clinically established disease stages.

Response:

I thank the reviewer for this important observation. I agree that the ordinal categories used in this study are **data-driven** and should not be interpreted as clinically established disease stages. Accordingly, I have revised the manuscript to consistently describe them as data-driven ordinal severity categories or quantile-derived ordinal categories throughout the revised manuscript. In the Abstract, Introduction, Results, Discussion, and Conclusion, I have removed wording that could imply clinically validated staging and explicitly clarified that these categories serve as an auxiliary modeling strategy to exploit the ordinal structure of continuous motor UPDRS scores in the absence of clinically annotated stage labels. I further acknowledge in the Discussion that future work will investigate clinically established staging criteria on larger longitudinal datasets. Please refer to the revised manuscript.

Sincerely,

K. A. Shahriar

Corresponding Author.
