# Supplementary material for: Uncertainty-aware personalized estimation of Parkinson’s disease severity from longitudinal speech
Source: PLoS One. 2026 Aug 5;21(8):e0343191. doi: 10.1371/journal.pone.0343191 (PMC13440870; doi:10.1371/journal.pone.0343191)
Supplement: Peer review history

# Peer review: Decision Letter 0

**DOI:** 10.1371/journal.pone.0343191.r001

**Authors:** Thiago P. Fernandes

15 May 2026

PONE-D-26-05550

Uncertainty-aware personalized estimation of Parkinson’s disease severity from longitudinal speech

PLOS One

Dear Dr. Shahriar,

Thank you for submitting your manuscript to PLOS ONE. After careful consideration, we feel that it has merit but does not fully meet PLOS ONE’s publication criteria as it currently stands. Therefore, we invite you to submit a revised version of the manuscript that addresses the points raised during the review process.

==============================

The reviewers recognize the conceptual relevance of the study and the clear formulation of the proposed uncertainty-aware framework. At the same time, several important concerns remain regarding generalizability, potential overfitting related to longitudinal subject structure, the role of patient-specific embeddings, and the interpretation and validation of the uncertainty estimates.

In particular, the exceptionally high predictive performance requires additional clarification and stronger validation. The revised version should more carefully address the possibility that performance may be partially driven by subject-specific structure or short-term temporal consistency rather than broader disease progression signals. Additional analyses, stronger baselines, ablation experiments, and a more cautious interpretation of the clinical implications would substantially strengthen the manuscript.

Please respond to all reviewer comments and highlight all changes in the revised ms.

==============================

Please submit your revised manuscript by Jun 29 2026 11:59PM. If you will need more time than this to complete your revisions, please reply to this message or contact the journal office at plosone@plos.org. When you're ready to submit your revision, log on to https://www.editorialmanager.com/pone/ and select the 'Submissions Needing Revision' folder to locate your manuscript file.

Please include the following items when submitting your revised manuscript:

- A letter that responds to each point raised by the academic editor and reviewer(s). You should upload this letter as a separate file labeled 'Response to Reviewers'.
- A marked-up copy of your manuscript that highlights changes made to the original version. You should upload this as a separate file labeled 'Revised Manuscript with Track Changes'.
- An unmarked version of your revised paper without tracked changes. You should upload this as a separate file labeled 'Manuscript'.

If you would like to make changes to your financial disclosure, please include your updated statement in your cover letter. Guidelines for resubmitting your figure files are available below the reviewer comments at the end of this letter.

If applicable, we recommend that you deposit your laboratory protocols in protocols.io to enhance the reproducibility of your results. Protocols.io assigns your protocol its own identifier (DOI) so that it can be cited independently in the future. For instructions see: https://journals.plos.org/plosone/s/submission-guidelines#loc-laboratory-protocols. Additionally, PLOS ONE offers an option for publishing peer-reviewed Lab Protocol articles, which describe protocols hosted on protocols.io. Read more information on sharing protocols at https://plos.org/protocols?utm_medium=editorial-email&utm_source=authorletters&utm_campaign=protocols.

As the corresponding author, your ORCID iD is verified in the submission system and will appear in the published article. PLOS supports the use of ORCID, and we encourage all coauthors to register for an ORCID iD and use it as well. Please encourage your coauthors to verify their ORCID iD within the submission system before final acceptance, as unverified ORCID iDs will not appear in the published article. *Only*  the individual author can complete the verification step; PLOS staff *cannot*  verify ORCID iDs on behalf of authors.

We look forward to receiving your revised manuscript.

Kind regards,

Thiago P. Fernandes, PhD

Academic Editor

PLOS One

Journal Requirements:

When submitting your revision, we need you to address these additional requirements.

1. Please ensure that your manuscript meets PLOS ONE's style requirements, including those for file naming. The PLOS ONE style templates can be found at https://journals.plos.org/plosone/s/file?id=wjVg/PLOSOne_formatting_sample_main_body.pdf and https://journals.plos.org/plosone/s/file?id=ba62/PLOSOne_formatting_sample_title_authors_affiliations.pdf

2. Please note that PLOS One has specific guidelines on code sharing for submissions in which author-generated code underpins the findings in the manuscript. In these cases, we expect all author-generated code to be made available without restrictions upon publication of the work. Please review our guidelines at https://journals.plos.org/plosone/s/materials-and-software-sharing#loc-sharing-code and ensure that your code is shared in a way that follows best practice and facilitates reproducibility and reuse.

If the reviewer comments include a recommendation to cite specific previously published works, please review and evaluate these publications to determine whether they are relevant and should be cited. There is no requirement to cite these works unless the editor has indicated otherwise.

[Note: HTML markup is below. Please do not edit.]

Reviewers' comments:

Reviewer's Responses to Questions

**Comments to the Author**

1. Is the manuscript technically sound, and do the data support the conclusions?

The manuscript must describe a technically sound piece of scientific research with data that supports the conclusions. Experiments must have been conducted rigorously, with appropriate controls, replication, and sample sizes. The conclusions must be drawn appropriately based on the data presented.

Reviewer #1: Yes

Reviewer #2: Partly

Reviewer #3: Yes

**********

2. Has the statistical analysis been performed appropriately and rigorously?

Reviewer #1: No

Reviewer #2: Yes

Reviewer #3: Yes

**********

3. Have the authors made all data underlying the findings in their manuscript fully available?

The PLOS Data policy requires authors to make all data underlying the findings described in their manuscript fully available without restriction, with rare exception (please refer to the Data Availability Statement in the manuscript PDF file). The data should be provided as part of the manuscript or its supporting information, or deposited to a public repository. For example, in addition to summary statistics, the data points behind means, medians and variance measures should be available. If there are restrictions on publicly sharing data—e.g. participant privacy or use of data from a third party—those must be specified.

Reviewer #1: Yes

Reviewer #2: Yes

Reviewer #3: Yes

**********

4. Is the manuscript presented in an intelligible fashion and written in standard English?

PLOS ONE does not copyedit accepted manuscripts, so the language in submitted articles must be clear, correct, and unambiguous. Any typographical or grammatical errors should be corrected at revision, so please note any specific errors here.

Reviewer #1: Yes

Reviewer #2: Yes

Reviewer #3: Yes

**********

5. Review Comments to the Author

Please use the space provided to explain your answers to the questions above. You may also include additional comments for the author, including concerns about dual publication, research ethics, or publication ethics. (Please upload your review as an attachment if it exceeds 20,000 characters)

Reviewer #1: The primary objective of this research is to develop a regression algorithm which is aware of uncertainty. This work assume noise in the PD -UPDRS scores. The strength of the paper are (i) clear articulation of the objective and logical mathematical discussions are clear.

The shortcomings are

1) Quantum of work is less

2)Its a popular dataset which consists of pre-calculated parameters and this work is another statistical interpretation.

3)In a bigger work this can be one way to substantiate the results and considering only this as a big work is not correct in my opinion. Thus the work is limited to a particular dataset and difficult to generalize.

4)Its Intellectually good piece of work but more contributions shall be done by considering a dataset with customized parameters of the authors choice and a full complete work shall be tried out. The noise shall be added synthetically to UPDRS and the robustness of the proposed algorithm shall be tested.

Reviewer #2: This manuscript presents an uncertainty-aware, personalized framework for estimating Parkinson’s disease severity from longitudinal speech data. The study addresses a clinically relevant problem, namely the need for scalable and objective telemonitoring tools, and proposes a conceptually interesting integration of temporal modeling, personalization, and probabilistic inference. The work is generally well written, methodologically ambitious, and supported by extensive experiments. However, several important methodological, interpretational, and validation issues limit the current strength of the conclusions. Addressing these concerns would substantially improve the robustness, transparency, and clinical relevance of the study.

Q1. Extremely high predictive performance and risk of overfitting

The reported performance is exceptionally high, particularly the near-perfect explained variance and very low prediction error. While the authors acknowledge dataset characteristics, these values raise concerns regarding potential overfitting or implicit leakage through temporal or subject-specific structure. The dataset consists of repeated longitudinal measurements with low short-term variability, which may inflate performance. The manuscript would benefit from a more critical and quantitative analysis of this issue. Specifically, please provide comparison with naive baselines such as last observation carried forward or subject-specific mean prediction, performance under more challenging splits such as reduced history or temporal extrapolation, and ablation of patient embeddings to assess how much performance depends on subject identity rather than disease signal.

Q2. Generalizability and external validity

The study relies on a single dataset with a relatively small cohort and a short observation period. All subjects are unmedicated and recently diagnosed, which significantly limits the generalizability of the findings. The conclusions regarding clinical applicability and telemonitoring potential are therefore somewhat overstated. At minimum, the manuscript should more explicitly acknowledge this limitation and temper claims accordingly. Ideally, validation on an independent cohort or at least a simulated domain shift analysis would strengthen the work.

Q3. Personalization strategy and potential information leakage

The use of learnable patient-specific embeddings is an interesting design choice, but it raises conceptual and practical concerns. Since embeddings are learned per subject, it is unclear how the model behaves for truly new patients in real-world deployment. Even though a patient-wise split is used, the mechanism by which embeddings are initialized or adapted for unseen patients is not sufficiently described. This is a critical issue, as personalization may inadvertently encode subject identity rather than disease progression. Please clarify how the model generalizes to new patients without prior embedding information and consider including an analysis without embeddings or with adaptation strategies.

Q4. Uncertainty quantification and clinical interpretation

The incorporation of uncertainty is a strength of the manuscript, but its clinical usefulness remains insufficiently demonstrated. The results suggest that uncertainty reflects variability but not necessarily prediction error. This limits interpretability in a clinical decision-making context. The manuscript would benefit from more rigorous evaluation of uncertainty calibration, for example using calibration curves, expected calibration error, or coverage probability of prediction intervals. Without such analysis, it is difficult to assess whether the uncertainty estimates are reliable.

Q5. Ordinal classification and clinical validity

The ordinal severity labels are derived using quantile-based thresholds rather than clinically established staging systems. While this approach is methodologically convenient, it reduces clinical interpretability and limits comparability with existing literature. The manuscript should discuss this limitation more explicitly and, if possible, provide an additional analysis using clinically meaningful thresholds or better justify the chosen discretization approach.

Q6. Comparison with prior work

Although a comparison with prior work is provided, it is not entirely fair due to differences in evaluation protocols, data splits, and preprocessing pipelines. Some prior works report substantially different performance under different conditions. While this is acknowledged, the manuscript still draws strong conclusions regarding superiority. The comparison should be framed more cautiously, and ideally, baseline models should be reimplemented and evaluated under the same experimental conditions.

Q7. Model complexity versus dataset size

The proposed model combines temporal modeling, latent variable inference, ordinal regression, and personalization. Given the relatively small dataset size, there is a risk that model complexity exceeds what the data can robustly support. The hyperparameter sensitivity analysis suggests stability, but does not fully address overparameterization. An ablation study demonstrating the contribution of each component would significantly strengthen the manuscript.

Q8. Clinical relevance and interpretation of results

While the methodological contribution is clear, the clinical implications are somewhat overstated. The manuscript suggests that the model could support clinical decision-making, yet no analysis is provided regarding clinically meaningful thresholds, progression detection, or treatment-related changes. The discussion should be more cautious and better aligned with what is actually demonstrated, namely predictive modeling on a specific dataset rather than validated clinical utility.

Q9. Dataset limitations and temporal scope

The dataset spans a relatively short period and shows weak progression trends at the population level. This raises questions about whether the model is truly capturing disease progression or primarily learning short-term temporal consistency and subject-specific baselines. The manuscript would benefit from a more explicit discussion of this limitation and its implications for long-term disease monitoring.

Q10. Minor issues and clarity

The manuscript is generally well written, but certain sections could be streamlined for clarity. Some methodological descriptions are dense and could benefit from simplification. A clearer schematic overview of the model architecture would also improve readability, particularly for readers less familiar with probabilistic modeling approaches.

Reviewer #3: This study aimed to build upon previous research using machine learning to detect altered speech patterns that can correlate to Parkinson’s disease severity by developing a new model that integrates longitudinal information, accounts for patient specific variability, quantifies uncertainty, and estimates both continuous UPDRS scores and ordinal severity categories. If appropriately developed, such a model could increase access to regular and objective evaluation of disease progression and could perhaps lead to predictive modeling for future disease severity. Overall, this is a well conducted study and clearly written manuscript. I just have several questions I would like addressed:

1. Limitations were noted with the current dataset including its short duration and including only unmedicated patients. Are there other independent datasets that could be used to validate the model, such as ones with medicated patients?

2. This model can predict UPDRS with longitudinal windows of 10 recordings. How well would the model perform with only one or two recordings? This would be important for real world use with new patients.

3. Could this model be adapted to predict future UPDRS scores?

4. The model is described as uncertainty aware and incorporates a variance estimate based on Gaussian distribution. While including a variance measure is useful and promising, it is not clear that the variance has been shown to correspond to clinically meaningful uncertainty. How should this variance be interpreted in the clinical setting? For example, does a wider uncertainty range indicate that the UPDRS estimate is less reliable?

5. Speech abnormalities are seen in other conditions. Could this impact the specificity of such a model.

6. Some acronyms were introduced without being expanded first. Please ensure all acronyms are defined.

**********

6. PLOS authors have the option to publish the peer review history of their article (what does this mean?). If published, this will include your full peer review and any attached files.

If you choose “no”, your identity will remain anonymous but your review may still be made public.

**Do you want your identity to be public for this peer review?**  For information about this choice, including consent withdrawal, please see our Privacy Policy.

Reviewer #1: No

Reviewer #2: **Yes:** Valentino Rački

Reviewer #3: No

**********

[NOTE: If reviewer comments were submitted as an attachment file, they will be attached to this email and accessible via the submission site. Please log into your account, locate the manuscript record, and check for the action link "View Attachments". If this link does not appear, there are no attachment files.]

To ensure your figures meet our technical requirements, please review our figure guidelines: https://journals.plos.org/plosone/s/figures

You may also use PLOS’s free figure tool, NAAS, to help you prepare publication quality figures: https://journals.plos.org/plosone/s/figures#loc-tools-for-figure-preparation.

NAAS will assess whether your figures meet our technical requirements by comparing each figure against our figure specifications.

# Author comment: Author response to Decision Letter 1

**DOI:** 10.1371/journal.pone.0343191.r002

8 Jun 2026

A detail reviewer's response is uploaded as a pdf file.

The responses are given below.

Response to the Reviewers

Reviewer 1

Comment 1: Quantum of work is less.

Response:

I thank the reviewer for this important observation. I agree that several individual components used in the proposed framework, such as recurrent temporal modeling and latent variable inference, have appeared separately in prior literature. However, the primary contribution of the present work lies in the integration of these components into a unified uncertainty-aware longitudinal telemonitoring framework for Parkinson’s disease severity estimation. In the revised manuscript, the evaluation criteria are made more rigorous, and several additional experiments are performed. Please refer to the revised manuscript.

Comment 2: Its a popular dataset which consists of pre-calculated parameters and this work is another statistical interpretation.

Response:

I thank the reviewer for this important observation. I acknowledge that the Oxford Parkinson’s telemonitoring dataset is a widely used benchmark dataset and that the acoustic dysphonia measures are precomputed rather than learned directly from raw speech signals. The primary objective of the present study is therefore not acoustic feature engineering or representation discovery, but rather the development and analysis of an uncertainty-aware longitudinal modeling framework for estimating Parkinson’s disease severity. The dataset was selected because it remains one of the few publicly available longitudinal Parkinson’s speech datasets containing repeated motor UPDRS assessments, enabling reproducible comparison with prior telemonitoring studies. I agree that reliance on precomputed features limits the scope of conclusions regarding low-level acoustic biomarker discovery, and I have clarified this limitation explicitly in the revised manuscript.

Comment 3: In a bigger work this can be one way to substantiate the results and considering only this as a big work is not correct in my opinion. Thus the work is limited to a particular dataset and difficult to generalize.

Response:

I thank the reviewer for this important observation and agree that evaluation on a single dataset limits the generalizability of the conclusions. The primary intentions of this present work are not to claim universal clinical applicability, but rather to investigate the feasibility of uncertainty-aware longitudinal modeling for Parkinson’s disease telemonitoring under controlled settings. I acknowledge that the Oxford Parkinson’s telemonitoring dataset represents a relatively small and specialized cohort with limited temporal duration and specific recording conditions. Accordingly, I have revised the manuscript to more carefully frame the proposed framework as a methodological proof-of-concept with experimental validations rather than a deployment-ready clinical system.

Comment 4: Its Intellectually good piece of work but more contributions shall be done by considering a dataset with customized parameters of the authors choice and a full complete work shall be tried out. The noise shall be added synthetically to UPDRS and the robustness of the proposed algorithm shall be tested.

Response:

I thank the reviewer for the constructive suggestion. I agree that validation on larger and more heterogeneous datasets, including datasets with customized or newly derived speech representations, would further strengthen the contribution and broader applicability of the proposed framework. However, the current work was primarily intended as a methodological study of uncertainty-aware longitudinal modeling using one of the few publicly available Parkinson’s telemonitoring datasets containing repeated motor UPDRS assessments.

To address the reviewer’s recommendation regarding robustness evaluation, I conducted additional experiments in which synthetic Gaussian noise was added to the motor UPDRS labels during training (Please refer to section 5.6 and Table 6). The results demonstrated gradual performance degradation as the noise level increased, while the framework retained relatively stable predictive behavior under moderate perturbation levels. These findings suggest that the proposed probabilistic longitudinal framework exhibits reasonable robustness to imperfect or noisy clinical supervision.

The revised manuscript now includes both quantitative results and a discussion of this robustness analysis, along with a more explicit acknowledgment of the limitations associated with the current dataset and feature representation (Please refer to the discussion section).

Reviewer 2

Comment 1: Extremely high predictive performance and risk of overfitting. The reported performance is exceptionally high, particularly the near-perfect explained variance and very low prediction error. While the authors acknowledge dataset characteristics, these values raise concerns regarding potential overfitting or implicit leakage through temporal or subject-specific structure. The dataset consists of repeated longitudinal measurements with low short-term variability, which may inflate performance. The manuscript would benefit from a more critical and quantitative analysis of this issue. Specifically, please provide comparison with naive baselines such as last observation carried forward or subject-specific mean prediction, performance under more challenging splits such as reduced history or temporal extrapolation, and ablation of patient embeddings to assess how much performance depends on subject identity rather than disease signal.

Response:

I thank the reviewer for this important and insightful observation. I agree that the initially reported predictive performance is exceptionally high and that the longitudinal structure of the dataset, including repeated measurements with relatively low short-term variability, may introduce strong temporal continuity and subject-specific effects that inflate performance. To address this concern more rigorously, I substantially expanded the experimental evaluation in the revised manuscript.

First, I added comparisons with several naive temporal baselines, including global mean prediction, subject-specific mean prediction, and last-observation-carried-forward (LOCF) (Please refer to Table.3 in the revised manuscript). The LOCF baseline achieved particularly strong performance, confirming the presence of substantial short-term temporal autocorrelation within the dataset trajectories. This analysis is now explicitly discussed in the manuscript to better contextualize the reported regression performance.

Second, I conducted additional temporal extrapolation experiments in which the model was trained on earlier trajectory segments and evaluated on later visits (Please refer to section 5.1, last paragraph). Under this substantially more challenging setting, predictive performance decreased compared to the original evaluation but remained reasonably strong, suggesting that the framework captures longer-range disease progression patterns beyond immediate temporal persistence.

Third, I performed limited-history experiments using temporal windows consisting of only one or two prior recordings to assess dependence on longitudinal context (Please refer to section 5.7). While performance improved with larger windows, the model retained stable predictive capability under sparse-history conditions, supporting potential applicability in realistic telemonitoring scenarios with limited prior data.

Finally, I added a component-wise ablation analysis, including the removal of patient-specific embeddings (Please refer to section 5.8). Results showed that patient embeddings contribute meaningfully to predictive performance, confirming that part of the model behavior depends on subject-specific longitudinal structure. This limitation is now discussed more explicitly in the revised manuscript.

Overall, the manuscript has been revised to more carefully position the proposed framework as an uncertainty-aware longitudinal telemonitoring model evaluated under multiple robustness analyses, rather than as a universally generalizable clinical prediction system.

Comment 2: Generalizability and external validity. The study relies on a single dataset with a relatively small cohort and a short observation period. All subjects are unmedicated and recently diagnosed, which significantly limits the generalizability of the findings. The conclusions regarding clinical applicability and telemonitoring potential are therefore somewhat overstated. At minimum, the manuscript should more explicitly acknowledge this limitation and temper claims accordingly. Ideally, validation on an independent cohort or at least a simulated domain shift analysis would strengthen the work.

Response:

I thank the reviewer for this important observation and agree that the use of a single relatively small longitudinal dataset limits the broader generalizability of the findings. The Oxford Parkinson’s telemonitoring dataset consists primarily of recently diagnosed and unmedicated patients recorded over a relatively short observation period, which does not fully represent the heterogeneity encountered in real-world clinical populations. Accordingly, I have revised the manuscript to moderate claims regarding clinical applicability and deployment readiness.

To address this concern more rigorously, I added additional robustness analyses in the revised manuscript. Specifically, I conducted temporal extrapolation experiments evaluating performance on later unseen trajectory segments, limited-history experiments using only one or two prior recordings, and a simulated domain shift analysis using synthetic perturbations of the clinical targets (Please refer to Table 6 in the revised manuscript). These experiments were intended to assess robustness under more challenging longitudinal conditions beyond the original evaluation setting.

While the proposed framework demonstrated reasonably stable behavior under these additional analyses, I agree that validation on larger independent multi-center cohorts with more heterogeneous disease stages, medication states, and recording conditions will be essential before broader clinical conclusions can be established. This limitation and the need for external validation are now discussed more explicitly in both the Discussion and Conclusion sections.

Comment 3: Personalization strategy and potential information leakage

The use of learnable patient-specific embeddings is an interesting design choice, but it raises conceptual and practical concerns. Since embeddings are learned per subject, it is unclear how the model behaves for truly new patients in real-world deployment. Even though a patient-wise split is used, the mechanism by which embeddings are initialized or adapted for unseen patients is not sufficiently described. This is a critical issue, as personalization may inadvertently encode subject identity rather than disease progression. Please clarify how the model generalizes to new patients without prior embedding information and consider including an analysis without embeddings or with adaptation strategies.

Response:

I thank the reviewer for this important observation. I agree that the use of patient-specific embeddings introduces important considerations regarding generalization to unseen subjects and the potential encoding of subject identity rather than disease-related dynamics. In the original manuscript, the role and limitations of the personalization mechanism were not sufficiently clarified, and this has now been revised.

In the current framework, embeddings are learned only for subjects observed during training and are not adapted online for unseen patients. Consequently, the personalized embeddings primarily function as subject-dependent latent context during longitudinal modeling rather than as a deployable patient initialization strategy for cold-start scenarios. To better assess the dependence of the framework on subject identity information, I conducted an additional ablation experiment in which patient embeddings were completely removed from the model (Please refer to section 5.8).

The ablation results showed a measurable reduction in predictive performance without embeddings, indicating that personalization contributes meaningfully to the learned longitudinal representation. However, the model remained functional even without patient-specific embeddings, suggesting that the temporal speech features themselves still contain predictive disease-related information beyond subject identity alone.

I agree that adaptation strategies for truly unseen patients represent an important future direction. The revised manuscript now discusses possible extensions, including meta-learning, adaptive embedding initialization, and online personalization strategies for real-world deployment settings (Please refer to the discussion section).

Comment 4: Uncertainty quantification and clinical interpretation

The incorporation of uncertainty is a strength of the manuscript, but its clinical usefulness remains insufficiently demonstrated. The results suggest that uncertainty reflects variability but not necessarily prediction error. This limits interpretability in a clinical decision-making context. The manuscript would benefit from more rigorous evaluation of uncertainty calibration, for example, using calibration curves, expected calibration error, or coverage probability of prediction intervals. Without such analysis, it is difficult to assess whether the uncertainty estimates are reliable.

Response:

I thank the reviewer for this important and constructive observation. I agree that visualizing uncertainty alone is insufficient to establish reliability in a clinical context and that more rigorous calibration analysis is necessary to interpret the uncertainty estimates meaningfully.

To address this concern, I added additional uncertainty calibration experiments in the revised manuscript, including prediction interval coverage probability (PICP) analysis and reliability calibration curves after variance scaling calibration (Please refer to section 5.5). The calibrated uncertainty estimates achieved coverage probabilities reasonably close to expected Gaussian confidence levels, indicating partially calibrated global predictive intervals. Reliability calibration analysis further demonstrated generally consistent calibration behavior across multiple confidence levels, although mild under-coverage remained at higher confidence intervals.

At the same time, correlation analysis between predictive uncertainty and sample-wise prediction error remained weak, suggesting that the current latent uncertainty formulation is more effective at providing globally calibrated confidence intervals than identifying locally difficult predictions. Accordingly, I revised the manuscript to moderate claims regarding clinical interpretability and clarified that the reported uncertainty should primarily be interpreted as model confidence rather than clinically validated predictive reliability (Please refer to discussion section).

I agree that more advanced uncertainty modeling and calibration strategies, including heteroscedastic likelihood modeling, conformal prediction, and Bayesian calibration techniques, represent important future directions for improving clinical applicability of uncertainty-aware telemonitoring systems.

Comment 5: Ordinal classification and clinical validity

The ordinal severity labels are derived using quantile-based thresholds rather than clinically established staging systems. While this approach is methodologically convenient, it reduces clinical interpretability and limits comparability with existing literature. The manuscript should discuss this limitation more explicitly and, if possible, provide an additional analysis using clinically meaningful thresholds or better justify the chosen discretization approach.

Response:

I thank the reviewer for this important observation. I agree that the ordinal severity labels used in this study were derived from quantile-based discretization rather than clinically established staging criteria, which limits direct clinical interpretability and comparability with prior clinical literature. The primary motivat

Attachment Submitted filename: Reviewers Comment.pdf

# Peer review: Decision Letter 1

**DOI:** 10.1371/journal.pone.0343191.r003

**Authors:** Thiago P. Fernandes

1 Jul 2026

PONE-D-26-05550R1

Uncertainty-aware personalized estimation of Parkinson’s disease severity from longitudinal speech

PLOS One

Dear Dr. Shahriar,

Thank you for submitting your manuscript to PLOS ONE. After careful consideration, we feel that it has merit but does not fully meet PLOS ONE’s publication criteria as it currently stands. Therefore, we invite you to submit a revised version of the manuscript that addresses the points raised during the review process.

Please submit your revised manuscript by Aug 15 2026 11:59PM. If you will need more time than this to complete your revisions, please reply to this message or contact the journal office at plosone@plos.org. When you're ready to submit your revision, log on to https://www.editorialmanager.com/pone/ and select the 'Submissions Needing Revision' folder to locate your manuscript file.

Please include the following items when submitting your revised manuscript:

- A letter that responds to each point raised by the academic editor and reviewer(s). You should upload this letter as a separate file labeled 'Response to Reviewers'.
- A marked-up copy of your manuscript that highlights changes made to the original version. You should upload this as a separate file labeled 'Revised Manuscript with Track Changes'.
- An unmarked version of your revised paper without tracked changes. You should upload this as a separate file labeled 'Manuscript'.

If you would like to make changes to your financial disclosure, please include your updated statement in your cover letter. Guidelines for resubmitting your figure files are available below the reviewer comments at the end of this letter.

If applicable, we recommend that you deposit your laboratory protocols in protocols.io to enhance the reproducibility of your results. Protocols.io assigns your protocol its own identifier (DOI) so that it can be cited independently in the future. For instructions see: https://journals.plos.org/plosone/s/submission-guidelines#loc-laboratory-protocols. Additionally, PLOS ONE offers an option for publishing peer-reviewed Lab Protocol articles, which describe protocols hosted on protocols.io. Read more information on sharing protocols at https://plos.org/protocols?utm_medium=editorial-email&utm_source=authorletters&utm_campaign=protocols.

As the corresponding author, your ORCID iD is verified in the submission system and will appear in the published article. PLOS supports the use of ORCID, and we encourage all coauthors to register for an ORCID iD and use it as well. Please encourage your coauthors to verify their ORCID iD within the submission system before final acceptance, as unverified ORCID iDs will not appear in the published article. *Only*  the individual author can complete the verification step; PLOS staff *cannot*  verify ORCID iDs on behalf of authors.

We look forward to receiving your revised manuscript.

Kind regards,

Thiago P. Fernandes, PhD

Academic Editor

PLOS One

Journal Requirements:

1. If the reviewer comments include a recommendation to cite specific previously published works, please review and evaluate these publications to determine whether they are relevant and should be cited. There is no requirement to cite these works unless the editor has indicated otherwise.

2. Please review your reference list to ensure that it is complete and correct. If you have cited papers that have been retracted, please include the rationale for doing so in the manuscript text, or remove these references and replace them with relevant current references. Any changes to the reference list should be mentioned in the rebuttal letter that accompanies your revised manuscript. If you need to cite a retracted article, indicate the article’s retracted status in the References list and also include a citation and full reference for the retraction notice.

Additional Editor Comments:

Please respond to all comments and highlight the changes in the revised ms.

[Note: HTML markup is below. Please do not edit.]

Reviewer's Responses to Questions

**Comments to the Author**

1. If the authors have adequately addressed your comments raised in a previous round of review and you feel that this manuscript is now acceptable for publication, you may indicate that here to bypass the “Comments to the Author” section, enter your conflict of interest statement in the “Confidential to Editor” section, and submit your "Accept" recommendation.

Reviewer #2: (No Response)

Reviewer #3: All comments have been addressed

**********

2. Is the manuscript technically sound, and do the data support the conclusions?

The manuscript must describe a technically sound piece of scientific research with data that supports the conclusions. Experiments must have been conducted rigorously, with appropriate controls, replication, and sample sizes. The conclusions must be drawn appropriately based on the data presented.

Reviewer #2: Yes

Reviewer #3: Yes

**********

3. Has the statistical analysis been performed appropriately and rigorously?

Reviewer #2: Yes

Reviewer #3: Yes

**********

4. Have the authors made all data underlying the findings in their manuscript fully available?

The PLOS Data policy requires authors to make all data underlying the findings described in their manuscript fully available without restriction, with rare exception (please refer to the Data Availability Statement in the manuscript PDF file). The data should be provided as part of the manuscript or its supporting information, or deposited to a public repository. For example, in addition to summary statistics, the data points behind means, medians and variance measures should be available. If there are restrictions on publicly sharing data—e.g. participant privacy or use of data from a third party—those must be specified.

Reviewer #2: Yes

Reviewer #3: Yes

**********

5. Is the manuscript presented in an intelligible fashion and written in standard English?

PLOS ONE does not copyedit accepted manuscripts, so the language in submitted articles must be clear, correct, and unambiguous. Any typographical or grammatical errors should be corrected at revision, so please note any specific errors here.

Reviewer #2: Yes

Reviewer #3: Yes

**********

6. Review Comments to the Author

Please use the space provided to explain your answers to the questions above. You may also include additional comments for the author, including concerns about dual publication, research ethics, or publication ethics. (Please upload your review as an attachment if it exceeds 20,000 characters)

Reviewer #2: I thank the author for the detailed response and for substantially revising the manuscript. In general, the author has addressed many of my previous concerns in a serious and constructive manner. The addition of naive temporal baselines, limited-history experiments, temporal extrapolation analysis, uncertainty calibration, synthetic label-noise robustness testing, and component-wise ablation analysis has strengthened the manuscript and provides a more balanced interpretation of the reported performance.

However, some concerns remain. First, although the additional analyses help contextualize the very high predictive performance, the reported R² remains extremely high and should continue to be interpreted cautiously. The manuscript should avoid implying that this level of performance would necessarily generalize to broader clinical settings.

Second, the issue of patient-specific embeddings is improved but not fully resolved. The author now clarifies that embeddings are learned for training subjects and are not adapted online for unseen patients. This is an important limitation for real-world deployment and should be emphasized more clearly, particularly in relation to cold-start use in new patients.

Third, the uncertainty analysis is improved, but the finding that uncertainty is weakly associated with sample-wise prediction error limits its clinical interpretability. The manuscript should clearly state that the current uncertainty estimates are not yet validated as reliable indicators of individual-level clinical prediction reliability.

Fourth, the ordinal severity classification remains based on quantile-derived categories rather than clinically validated staging thresholds. This is acceptable as an auxiliary modeling strategy, but the abstract and discussion should avoid presenting these categories as clinically established disease stages.

Overall, the revised manuscript is substantially improved and most of my concerns have been addressed at least in part. I would support consideration after minor revision, provided that the authors further temper claims regarding clinical applicability, telemonitoring readiness, and uncertainty-guided clinical decision-making.

Reviewer #3: (No Response)

**********

7. PLOS authors have the option to publish the peer review history of their article (what does this mean?). If published, this will include your full peer review and any attached files.

If you choose “no”, your identity will remain anonymous but your review may still be made public.

**Do you want your identity to be public for this peer review?**  For information about this choice, including consent withdrawal, please see our Privacy Policy.

Reviewer #2: No

Reviewer #3: No

**********

[NOTE: If reviewer comments were submitted as an attachment file, they will be attached to this email and accessible via the submission site. Please log into your account, locate the manuscript record, and check for the action link "View Attachments". If this link does not appear, there are no attachment files.]

To ensure your figures meet our technical requirements, please review our figure guidelines: https://journals.plos.org/plosone/s/figures

You may also use PLOS’s free figure tool, NAAS, to help you prepare publication quality figures: https://journals.plos.org/plosone/s/figures#loc-tools-for-figure-preparation.

NAAS will assess whether your figures meet our technical requirements by comparing each figure against our figure specifications.

# Author comment: Author response to Decision Letter 2

**DOI:** 10.1371/journal.pone.0343191.r004

6 Jul 2026

N/A. a detailed response is attached as PDF

Attachment Submitted filename: Response to the Reviewer.pdf

# Editor report: Decision Letter 2

**DOI:** 10.1371/journal.pone.0343191.r005

**Authors:** Thiago P. Fernandes

20 Jul 2026

Uncertainty-aware personalized estimation of Parkinson’s disease severity from longitudinal speech

PONE-D-26-05550R2

Dear Dr. Shahriar,

We’re pleased to inform you that your manuscript has been judged scientifically suitable for publication and will be formally accepted for publication once it meets all outstanding technical requirements.

Within one week, you’ll receive an e-mail detailing the required amendments. When these have been addressed, you’ll receive a formal acceptance letter and your manuscript will be scheduled for publication.

An invoice will be generated when your article is formally accepted. Please note, if your institution has a publishing partnership with PLOS and your article meets the relevant criteria, all or part of your publication costs will be covered. Please make sure your user information is up-to-date by logging into Editorial Manager at Editorial Manager® and clicking the ‘Update My Information' link at the top of the page. For questions related to billing, please contact billing support.

If your institution or institutions have a press office, please notify them about your upcoming paper to help maximize its impact. If they’ll be preparing press materials, please inform our press team as soon as possible -- no later than 48 hours after receiving the formal acceptance. Your manuscript will remain under strict press embargo until 2 pm Eastern Time on the date of publication. For more information, please contact onepress@plos.org.

Kind regards,

Thiago P. Fernandes, PhD

Academic Editor

PLOS One

Additional Editor Comments (optional):

Reviewers' comments:

# Editor report: Acceptance letter

**DOI:** 10.1371/journal.pone.0343191.r006

**Authors:** Thiago P. Fernandes

PONE-D-26-05550R2

PLOS One

Dear Dr. Shahriar,

I'm pleased to inform you that your manuscript has been deemed suitable for publication in PLOS One. Congratulations! Your manuscript is now being handed over to our production team.

At this stage, our production department will prepare your paper for publication. This includes ensuring the following:

* All references, tables, and figures are properly cited

* All relevant supporting information is included in the manuscript submission,

* There are no issues that prevent the paper from being properly typeset

You will receive further instructions from the production team, including instructions on how to review your proof when it is ready. Please keep in mind that we are working through a large volume of accepted articles, so please give us a few days to review your paper and let you know the next and final steps.

Lastly, if your institution or institutions have a press office, please let them know about your upcoming paper now to help maximize its impact. If they'll be preparing press materials, please inform our press team within the next 48 hours. Your manuscript will remain under strict press embargo until 2 pm Eastern Time on the date of publication. For more information, please contact onepress@plos.org.

You will receive an invoice from PLOS for your publication fee after your manuscript has reached the completed accept phase. If you receive an email requesting payment before acceptance or for any other service, this may be a phishing scheme. Learn how to identify phishing emails and protect your accounts at https://explore.plos.org/phishing.

If we can help with anything else, please email us at customercare@plos.org.

Thank you for submitting your work to PLOS ONE and supporting open access.

Kind regards,

PLOS ONE Editorial Office Staff

on behalf of

Dr. Thiago P. Fernandes

Academic Editor

PLOS One
